# Stress Activated Protein Kinase Pathway Modulates Homologous Recombination in Fission Yeast

**DOI:** 10.1371/journal.pone.0047987

**Published:** 2012-10-31

**Authors:** Angela Bellini, Pierre-Marie Girard, Sarah Lambert, Ludovic Tessier, Evelyne Sage, Stefania Francesconi

**Affiliations:** 1 Institut Curie, Centre de Recherche, Orsay, France; 2 CNRS UMR 3348, Centre Universitaire, Orsay, France; Universita' di Milano, Italy

## Abstract

Rad52 is a key player in homologous recombination (HR), a DNA repair pathway that is dedicated to double strand breaks repair and recovery of perturbed replication forks. Here we show that fission yeast Rad52 homologue is phosphorylated when S phase cells are exposed to ROS inducers such as ultraviolet A radiation or hydrogen peroxide, but not to ultraviolet C or camptothecin. Phosphorylation does not depend on kinases Chk1, Rad3, Tel1 or Cdc2, but depends on a functional stress activated protein kinase (SAPK) pathway and can be partially prevented by anti-oxidant treatment. Indeed, cells lacking Sty1, the major fission yeast MAP kinase of the SAPK pathway, do not display Rad52 phosphorylation and have UVA induced Rad52 foci that persist longer if compared to wild type cells. In addition, spontaneous intrachromosomal HR is diminished in cells lacking Sty1 and, more precisely, gene conversion is affected. Moreover, HR induced by site-specific arrest of replication forks is twice less efficient in cells that do not express Sty1. Importantly, impairing HR by deletion of the gene encoding the recombinase Rhp51 leads to Sty1 dependent Rad52 phosphorylation. Thus, SAPK pathway impinges on early step of HR through phosphorylation of Rad52 in cells challenged by oxidative stress or lacking Rhp51 and is required to promote spontaneous gene conversion and recovery from blocked replication forks.

## Introduction

UVA radiation is the most abundant solar UV radiation that reaches earth's surface. UVA is able to penetrate human skin deeper than UVB and reaches the basal layer of skin where actively replicating keratinocytes are present. Different from UVB, UVA is weakly absorbed by DNA and mainly acts through interaction with endogenous photosensitizers resulting in generation of reactive oxygen species (ROS), predominantly singlet oxygen, which can damage all cellular components [1], [2], [3]. UVA induced ROS have been linked to skin photoaging [4] and increasing evidence suggest contribution to skin carcinogenesis [5].

More generally, ROS-induced cellular damage is linked to pathological conditions such as cancer, diabetes, atherosclerosis, neurodegenerative diseases and premature aging [6], [7], [8], [9], [10]. Because UVA induced biological effects are oxygen dependent, UVA is an inducer of oxidative stress and thus, cellular response to this radiation is complex. As many stresses, UVA-induced oxidative stress activates the SAPK pathways that are characterized by a cascade of kinases highly conserved: MAPK (mitogen activated protein kinase) kinase kinases (MAPKKK), MAPK kinases (MAPKK) and MAPKs. Eventually, activation of SAPK pathways results in protection of cells from injuries through appropriate regulation of gene expression and protein translation [11], [12]. In human keratinocytes the p38 MAP kinase and JNK (c-Jun N-terminal kinase) pathways are activated by UVA radiation [13].

In *Schizosaccharomyces pombe* (*S. pombe*), Sty1/Spc1 is the principal MAPK, mainly related to human p38, which is activated by phosphorylation following a variety of stresses including high osmolarity, oxidative stress, UVC exposure [14], [15] and metabolic inputs such as calorie restriction and histidine starvation, two situations generating endogenous oxidative stress [16], [17]. Sty1 is also required for sexual differentiation by regulating Ste11 expression, the major transcription factor required to induce meiotic genes [18]. Activation of Sty1 results in its nuclear localization and induction of gene expression mainly through the Atf1 transcription factor. Such modulation of the gene expression program will provide cells with the necessary to face stress [19]. Furthermore, Sty1, but not its major target Atf1, controls mitotic entry upon nutritional stress [20], [21]. However, different degrees of Sty1 activation impact on mitotic commitment in opposite ways: basal level of Sty1 activity promotes mitotic onset while high level of Sty1 activity delays mitotic entry [22]. This function is also conserved in mammals where the extracellular signal-related kinase (ERK) is required for mitotic entry and p38 activation by stress is required for mitotic delay [23], [24], [25]. In addition, different ways of Sty1 activation according to the stimulus applied have been reported, implying that Sty1, similarly to mammalian SAPKs, is finely tuned in the cell [26], [27], [28].

Nowadays, it is well established that UVA solar radiation is a biological relevant genotoxic agent [29]. At the DNA level UVA induces formation of oxidized bases, mainly 8-oxoguanine (8-oxoG), cyclobutane pyrimidine dimers (CPDs), pyrimidine (6-4) pyrimidone photoproducts (6-4PPs), single strand breaks and DNA protein cross-linking [2]. 8-oxoguanine DNA glycosylase OGG1, which removes 8-oxoG, efficiently prevents UVA-induced mutagenesis in yeast *Saccharomyces cerevisiae* [30].

HR is a DNA repair pathway dedicated to DSBs repair and recovery of blocked replication forks. Failure to repair DSBs results in cell death while inaccurate repair results in genome instability. HR must be tightly regulated to avoid dangerous outcomes that challenge genome stability and to prevent accumulation of DNA structures that are toxic for the cell [31]. At stalled replication forks HR allows to resume replication following fork collapse, but this mechanism can potentially induce gross chromosomal rearrangements and thus genomic instability [32].

*S. pombe* encodes a Rad52 homologue called spRad22. Here we will refer to the fission yeast protein as Rad52. This protein, a so-called mediator protein central to HR, is essential for both Rad51 (spRhp51) dependent and independent DSB repair pathways [33], [34]. Rad52 is required to replace Replication protein A (RPA) bound to single stranded DNA (ssDNA) by Rad51 (called Rhp51 in fission yeast), which will in turn promote homology search and D-loop formation [35]. However, Rad52 can promote HR in a Rad51 independent way because of its ability to anneal complementary single stranded DNA [36].

Recombination proteins, including Rad52, localize at double strand breaks to form discrete foci that occurs either spontaneously in S phase cells or after induction of DNA damage. In budding and fission yeast, Rad52 foci formation is a marker of ongoing HR [37], [38].

We previously reported that, in both mammals and fission yeast, DNA replication is perturbed by UVA exposure and that, despite activation of checkpoint pathways, UVA induced delay in DNA synthesis is largely checkpoint and Sty1 independent [39], [40]. In addition, we have shown that cells lacking Sty1 are UVA sensitive, indicating that the SAPK pathway plays an important role in response to this radiation also in fission yeast. Furthermore, cells exposed to UVA during S phase accumulate HR foci and this DNA repair pathway is required for survival to UVA [39].

We sought to further investigate on the interplay between HR and SAPK pathways in response to oxidative stress using UVA and hydrogen peroxide (H_2_O_2_). Here we report that Rad52 is phosphorylated in cells exposed to exogenous oxidative stress or in cells lacking Rhp51 and that phosphorylation is dependent on efficient SAPK pathway. Disabling the SAPK pathway delays resolution of UVA-induced Rad52 foci, affects spontaneous HR and HR occurring at blocked replication forks.

## Materials and Methods

### Yeast strains, media, growth conditions

The strains used in this study are listed in Table 1. Standard techniques were used for yeast growth and strains construction. Strains were grown in YE-rich medium (DIFCO) containing 2% glucose and supplemented with adenine, leucine, uracile, arginine and histidine [41]. Strains containing the RuraR substrate were grown in EMM glutamate (MP Biomedicals) medium with or without thiamine as described in [32].

**Table 1.Strains:** used in this study.

STRAIN	GENOTYPE	SOURCE
*rad52YFP*	*h- rad22YFP:KanR*	[37]
*chk1-d rad52YFP*	*h- rad22YFP:KanR chk1::ura4+ ura4-D18 ade6-M216*	This study
*cdc2-33ts rad52YFP*	*h- rad22YFP:KanR cdc2-3ts ura4-D18*	This study
*rad3-d rad52YFP*	*h+ rad3::ura4+ rad22:YFP:KanR leu1-32 ura4-D18*	This study
*tel1-d rad52YFP*	*h- tel1::KanR rad22:YFP:KanR leu1-32 ura4-D18*	This study
*sty1-d rad52YFP*	*h+ rad22YFP:KanR sty1::ura4+ ura4-D18 leu1-32 ade6-M216*	This study
*srr2-d rad52YFP*	*h- rad22YFP:kanR srr2::kanR*	This study
*sty1+ (MCW429)*	*h+ ura4-D18 leu1-32 his3-D1 arg3-D4 ade6- M375 int:: pUC8/his3+/ade6- L469*	[34]
*sty1-d*	*h+ ura4-D18 leu1-32 his3-D1 arg3-D4 ade6-L469/pUC8/his3+/ade6-M375 sty1::KanR*	This study
*RuraR sty1+*	*h+ sup35::nmt41::rtf1 RuraR ade6-704 leu1-32*	[32]
*RuraR rad52-d*	*h+ nmt41::rtf1:: sup35 ade6-704 leu1-32 RuraR rad22::KanR*	[32]
*RuraR sty1-d*	*h+ nmt41::rtf1:: sup35 ade6-704 leu1-32 RuraR sty1::NatR*	This study
*rhp51-d rad52YFP*	*h+ rhp51::ura4+ ura4-D18 rad22YFP:kanR*	This study
*rhp51-d sty1-d rad52YFP*	*h+ rhp51::ura4+ sty::ura4+ ura4-D18 rad22YFP:KanR leu1-32*	This study

### Genotoxic, oxidative and anti-oxidant treatments

Synchronization in early S phase was achieved by 4 hours treatment with 12 mM hydroxyurea (HU) (Sigma). Cells were then collected, resuspended in H_2_0 and UVA irradiated as described in [39]. UVC irradiation was achieved using StratalinkerTM (Stratagene) as described in [42]. Camptothecin (CPT) (Sigma) and H_2_0_2_ treatment (Sigma) was performed by releasing HU synchronized cells into fresh medium containing the chemical at the indicated concentrations. Anti-oxidant treatment was achieved adding 30 mM of N-acetylcysteine (NAC) (Sigma).

### SDS-PAGE, immunoblot and phosphatase treatment

Protein extracts were done according to [42]. To analyze Rad52, 80 mg (unless differently stated) of each protein extract was separated by electrophoresis at 40 Volts over-night on 7.5% acrylamide SDS-PAGE (acrylamide : bis-acrylamide 37.5: 1) using the STURDIER vertical SE 400 gel unit (Hoefer Scientific Instruments). Proteins were transferred for 2 hours at 120 Volts on nitrocellulose membrane (PROTRAN Whatman) using a Biorad Trans-Blot® Cell system. Membranes were probed with mouse anti-GFP antibody (Roche).

Equal amount of protein extracts were incubated for 1 hour at 30°C with or without **l** phosphatase (New England Biolabs) according to manufactory instructions.

### Microscopy and flow cytometry

Percentage of cells with Rad52 foci was scored on microphotographs as described in [37]. DNA content was analyzed by staining fixed cells with sytox green (Invitrogen) followed by flow cytometry analysis with FACSCalibur flow cytometer (Becton Dickinson). Data were plotted using CellQuest software. Percentage of cells with 2 nuclei was scored by staining fixed cells with DAPI (4′,6-diamidino-2-phenylindole, Sigma).

Intracellular peroxide levels were measured as described in [16]. Breafly, 1 ml of exponentially growing cells was incubated with 30 mM DHR123 (Dihydrorhodamine 123, Invitrogen) and 4.4 mM PI (Propidium Iodide, Sigma) for 30 minutes in the dark at 30°C. ROS production (DHR123) and dead cells (PI) were simultaneously analyzed using a FACSCalibur flow cytometer. ROS levels of unstained PI cells (living cells) were normalized to cell size.

### Fluctuation test and HR rate estimation

Fluctuation test was done as follow: 9 Ade- His+ colonies were independently inoculated in 10 ml of YE-rich medium and incubated at 30°C with agitation till cultures were around 5×10^7^ cells/ml for reasons explained in the text. Cells were plated on YE-rich medium to estimate viability. About 3×10^5^ cells were plated on EMM medium lacking adenine to estimate the frequency of Ade+ recombinants. Plates were replicated on EMM lacking adenine and histidine to estimate frequency of Ade+ His+ recombinants. Frequencies were analyzed by MSS-MLE (Ma-Sandri-Sarkar Maximum Likelihood Estimator) method with the program FALCOR (Fluctuation AnaLysis CalculatOR) to estimate the rate of recombination [43]. For each strain at least three independent experiments of nine cultures each were performed.

### RuraR assay, RFLA and PFGE

Procedure for RFLA (Restriction Fragment Length Analysis) to determine % of recombination at blocked replication forks and PFGE (Pulse Field Gel Electrophoresis) are extensively described in [44], [45]. Briefly, one single colony of each strain was grown in 10 ml of EMM + thiamine (OFF) for 24 hours at 30°C. Cells are then washed twice and inoculate in EMM + thiamine (OFF) and EMM - thiamine (ON) for 24 hours. Recombination between the RTS1 inverted repeats is detected by Southern blot on genomic DNA digested either with Ase I or with EcorV restriction enzyme using the *ura4* probe. % of recombination was estimated on the basis of three independent experiments. Analysis of acentric chromosome III by PFGE was done using *rng3* probe.

## Results

### Rad52 is phosphorylated in cells undergoing S phase upon UVA exposure

We have previously shown that cells irradiated with UVA in early S phase delay DNA replication and accumulate Rad52 foci, a marker of HR, in a dose dependent manner. We also showed that Rad52 is required for survival of cells irradiated in S phase, indicating that HR is necessary to repair some of the DNA lesions induced by UVA radiations [39].

We took advantage of the strain *rad52YFP*, which expresses the Rad52 protein tagged in C-terminal with YFP (Yellow Fuorescent Protein) [37], to analyze potential post-translational modifications of Rad52 protein by immunoblot with anti-GFP antibodies in cells exposed to UVA in early S phase. This strain was previously shown to delay DNA replication upon UVA exposure in early S phase similarly to *wt* cells [39].

**Figure 1:**
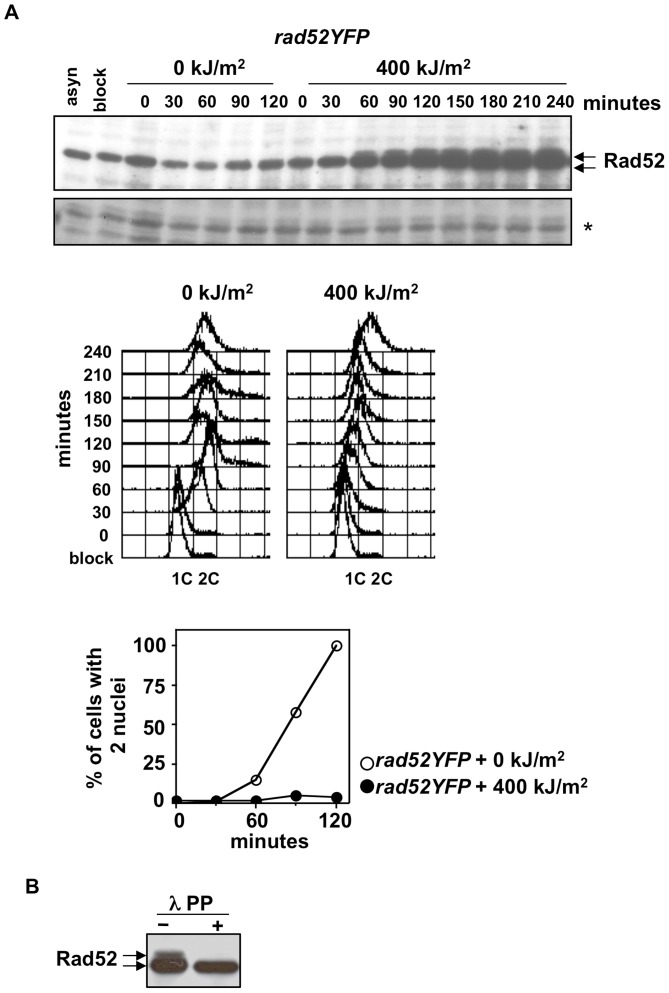
Rad52 is phosphorylated in S phase cells exposed to UVA radiations. (**A**) Protein extracts analyzed by immunoblot with anti-GFP antibodies (upper panel) to detect Rad52 protein from *rad52YFP* cells synchronized in early S phase by HU (block), collected in water and released in fresh medium after 0 or 400 kJ/m^2^ UVA for different time points. “asyn” indicates cells in log phase. Star refers to the same membrane colored with Rouge Ponceau to serve as loading control. An aliquot of cells at different time points was used to estimate cell cycle progression by FACS analysis and by scoring the percentage of cells that passed mitosis (cells with 2 nuclei). 1C and 2C indicate DNA content. (**B**) The change in mobility shift of Rad52 protein upon UVA is abolished by treatment with l phosphatase (PP).

Cells were synchronized in early S phase by HU treatment and released into cell cycle after UVA irradiation at 0 or 400 kJ/m^2^. Cell cycle progression was followed by FACS analysis and by staining cells with DAPI in order to monitor the number of cells that have passed mitosis (cells with 2 nuclei). Protein extracts were analyzed from cells prior synchronization (asyn), after 4 hours of HU treatment (block), and every 30 minutes after release into cell cycle from time 0 (immediately after irradiation) till 240 minutes. In non irradiated cells a single band corresponding to Rad52-YFP (referred to as Rad52) was detected at all time points, while in irradiated cells, an additional Rad52 slower migrating band was detected after 60 minutes from release (Figure 1A upper panel), time at which cells are progressing through S phase as shown by FACS analysis (Figure 1A middle panel) and haven't yet passed into mitosis (Figure 1A lower panel). It is to note that Rad52 protein levels seem to increase in irradiated cells. Treatment with l phosphatase (l PP) of a protein sample from UVA irradiated cells abolished the slower migrating band, indicating that it results from phosphorylation (Figure 1B).

Thus, this experiment shows that Rad52 is phosphorylated in response to UVA radiation.

### Rad52 phosphorylation is observed upon oxidative stress, but not after UVC or CPT treatment

In order to establish if Rad52 phosphorylation is specific to UVA treatment, we examined it in S phase cells exposed to different DNA damaging agents such as UVC radiation and CPT.

**Figure 2:**
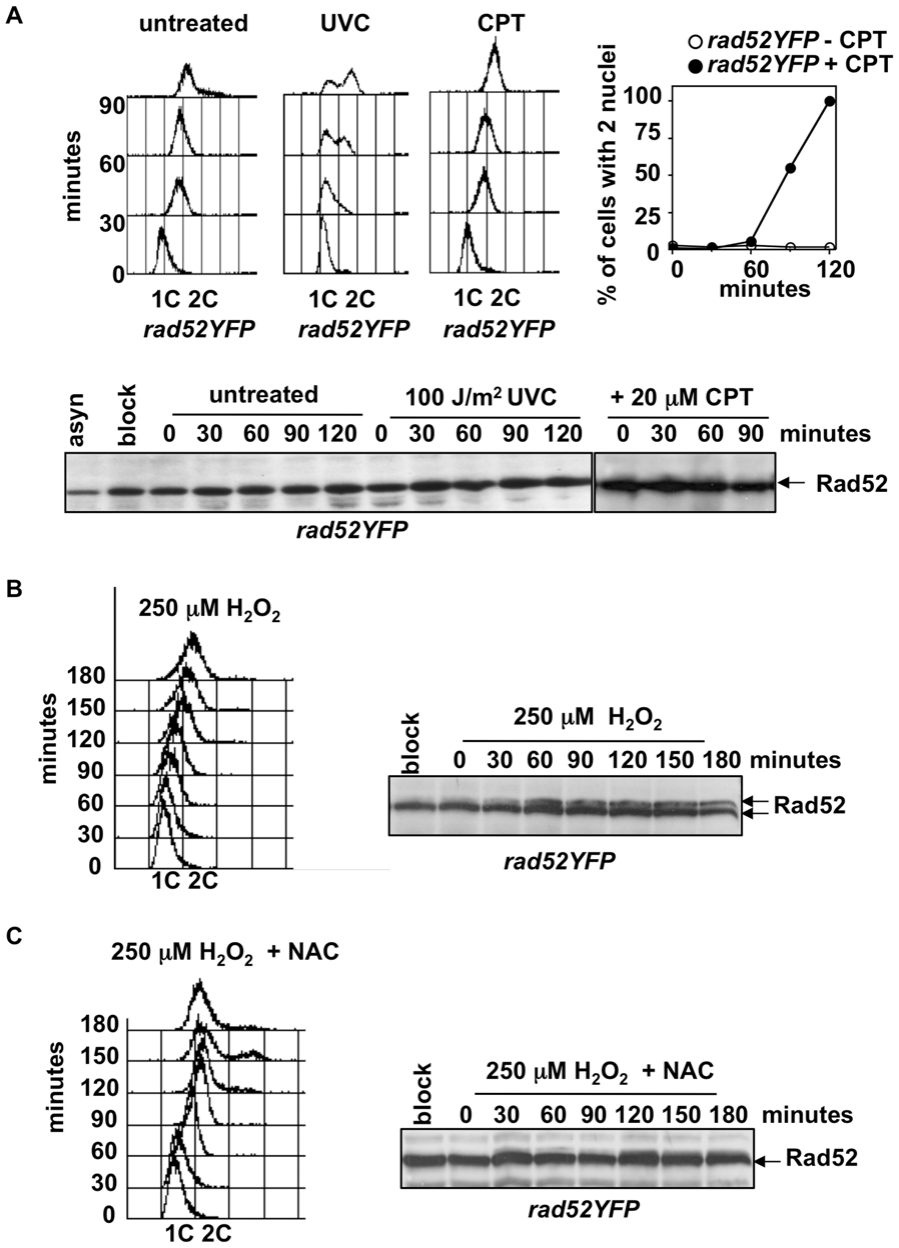
H_2_O_2_, but not UVC radiation or CPT, induces Rad52 phosphorylation. (**A**) FACS analysis (left panel) of *rad52YFP* cells synchronized in early S phase by HU (block), collected in water and released into fresh medium either without treatment (untreated), or after exposure to 100 J/m^2^ of UVC radiation, or in the presence of 20 mM of CPT. 1C and 2C indicate DNA content. Percentage of cells with 2 nuclei scored in untreated and CPT treated cells shows the delay in mitotic entry imposed by the treatment (right panel). Time 0 is immediately after release into cell cycle in presence or not of CPT. Aliquots of cells at different time points were processed for protein extracts that were analyzed by immunoblot with anti-GFP antibodies to detect Rad52 protein. (**B**) Cell cycle progression and Rad52 phosphorylation at different time points of *rad52YFP* cells synchronized in early S phase (block) and released into cell cycle in the presence of 250 mM of H_2_O_2_. (**C**) Cell cycle progression and Rad52 detection at different time points of *rad52YFP* cells synchronized in early S phase (block) and released into cell cycle in the presence of 250 mM of H_2_O_2_ and 30 mM of anti-oxidant (+ NAC).

UVC radiation directly damages DNA producing mainly CPDs that block replication fork progression [46]. Thus, we analyzed the Rad52 protein in cells synchronized in early S phase and exposed to 100 J/m^2^ UVC radiation prior release into cell cycle. DNA replication progression was followed every 30 minutes by FACS analysis. Time 0 corresponds to the moment where cells were released into cell cycle immediately after irradiation. As expected, DNA replication was delayed in UVC treated cells if compared to untreated ones (Figure 2A). However, despite delaying S phase, UVC irradiated cells did not display the slower migrating form of Rad52 indicating that UVC radiation does not induce detectable Rad52 phosphorylation (Figure 2A).

CPT is a topoisomerase I inhibitor that, at the concentration of 20 mM, generates DSBs in S phase cells and formation of Rad52 foci. Indeed, similarly to UVA [39], HR is required for cell survival to CPT treatment and cells activate Chk1 kinase to delay G2/M transition [47], [48], [49]. Thus, we analyzed S phase progression and Rad52 phosphorylation in HU synchronized cells released into cell cycle in the presence of 20 mM CPT. Under these conditions and in contrast to UVA and UVC, S phase progression was not delayed as judged by FACS analysis (Figure 2A) but, as expected, entry into mitosis was delayed by the treatment as judged by the persistence of cells with one nucleus in CPT treated culture (Figure 2A). However, similarly to UVC exposure, Rad52 phosphorylation was not detected (Figure 2A).

Then, we asked if an oxidative agent different from UVA would induce replication delay and Rad52 phosphorylation, and if both would depend on the presence of ROS. Thus, cells synchronized in early S phase were released into cell cycle in the presence of 250 mM of H_2_O_2_ or in the presence of H_2_O_2_ and 30 mM of NAC, an anti-oxidant able to counterbalance H_2_O_2_ effects. Cell cycle progression and Rad52 phosphorylation were assessed at different time points by FACS analysis and western blot, respectively. In cells exposed to H_2_O_2_ DNA replication was delayed (compare FACS panel in figure 2B to FACS panel “untreated” in figure 2A) and Rad52 protein was phosphorylated starting at 30 minutes from release (Figure 2B). When cells were released into cell cycle in presence of both oxidant and NAC, cell cycle delay was diminished if compared to cell exposed only to H_2_O_2_, although it was not completely abolished. Indeed, in cells treated with H_2_O_2_ the first round of replication ended around 150 minutes post release (Figure 2B), in the presence of H_2_O_2_ and NAC it was completed at 90 minutes (Figure 2C), while in untreated cells DNA replication ended around 60 minutes (Figure 2A “untreated”). Furthermore, Rad52 phosphorylation was barely visible in cells exposed to H_2_O_2_ along with NAC (Figure 2C).

In conclusion, UVC and CPT did not induce Rad52 phosphorylation, while treatment with 250 mM H_2_O_2_ resulted in DNA replication slow down and Rad52 modification, as for UVA radiation. Furthermore, NAC prevented in part both cell cycle slow down and Rad52 phosphorylation, pointing to the possibility that both events rely on ROS.

### Rad52 phosphorylation is Sty1 dependent

**Figure 3:**
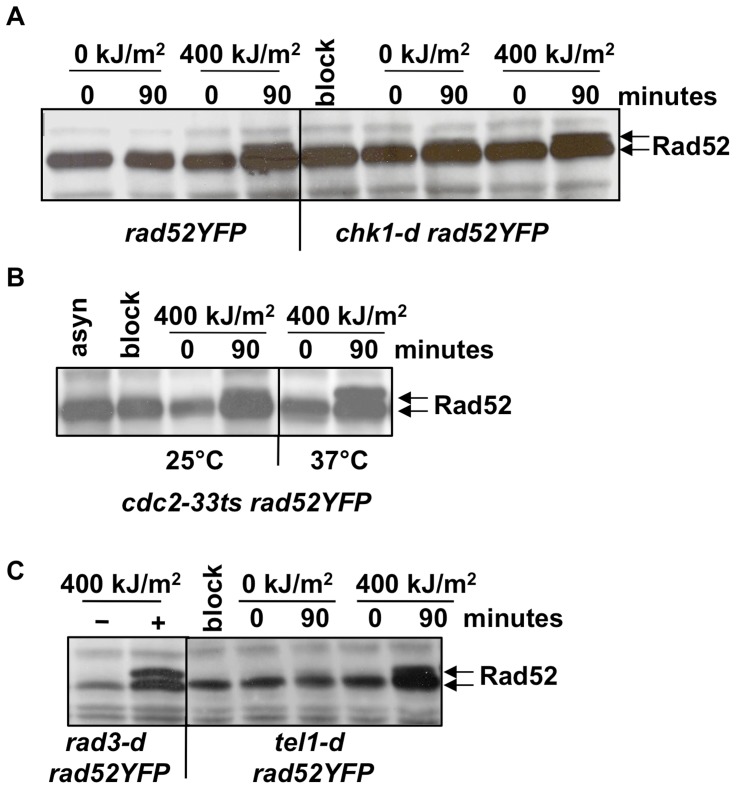
Rad52 phosphorylation upon UVA is independent of Chk1, Cdc2, Rad3 and Tel1 kinases. (**A**) Cells expressing or not the Chk1 kinase were irradiated with either 0 or 400 kJ/m^2^ UVA after synchronization in early S phase and Rad52 protein was detected by immunoblot with anti-GFP antibodies at 0 and 90 minutes after release. (**B**) Rad52 protein was detected at the indicated time points in the *cdc2-33ts* strain synchronized by HU treatment at permissive temperature (25°C) and released into cell cycle upon UVA radiation either at permissive or non permissive (37°C) temperature. (**C**) Rad52 protein detected in *rad3-d* asynchronous cells after exposition (+) or not (−) to UVA radiation and in *tel1-d* cells synchronized by HU treatment (block) and released into cell cycle for 0 and 90 minutes after treatment with either 0 or 400 kJ/m^2^ UVA.

We previously showed that checkpoint Chk1 kinase is phosphorylated in S phase cells irradiated with UVA [39]. We asked if Chk1 would be responsible for the Rad52 phosphorylation observed in UVA irradiated cells. Cultures of wild-type (*rad52YFP*) and *chk1* null mutant cells (*chk1-d rad52YFP*) where synchronized by HU in early S phase, exposed to 400 kJ/m^2^ and release into cell cycle. Rad52 protein was analyzed after HU treatment (block) and at 0 and 90 minutes after release. As shown in Figure 3A, Rad52 phosphorylation was detected in both strains, indicating that Chk1 kinase is not required for Rad52 modification.

We questioned if the Cdc2 kinase, a major cell cycle regulator, would be implicated in Rad52 phosphorylation. To do that we took advantage of the thermosensitive allele *cdc2-33* [50]. Cells synchronized in early S phase were released into cell cycle after UVA irradiation either at permissive temperature of 25°C, or at non permissive temperature of 37°C. Rad52 phosphorylation was assessed immediately after release (time 0) and after 90 minutes (Figure 3B). At both temperatures Rad52 phosphorylation was detected indicating that Cdc2 is unlikely the kinase required for such modification.

We then asked if Rad3 (ATR), the main upstream kinase in DNA damage checkpoint, or Tel1 (ATM) kinases were involved in Rad52 phosphorylation. Because cells lacking Rad3 (*rad3-d*) cannot be synchronized by HU in early S phase, we used asynchronous cultures to assess the phosphorylation state of Rad52 in this genetic background. Differently, cells lacking Tel1 (*tel1-d*) were synchronized by HU and released into cell cycle after exposure to either 0 or 400 kJ/m^2^ UVA. We found that upon UVA exposure Rad52 phosphorylation is still detected in both *rad3-d* and *tel1-d* cells (Figure 3C).

**Figure 4:**
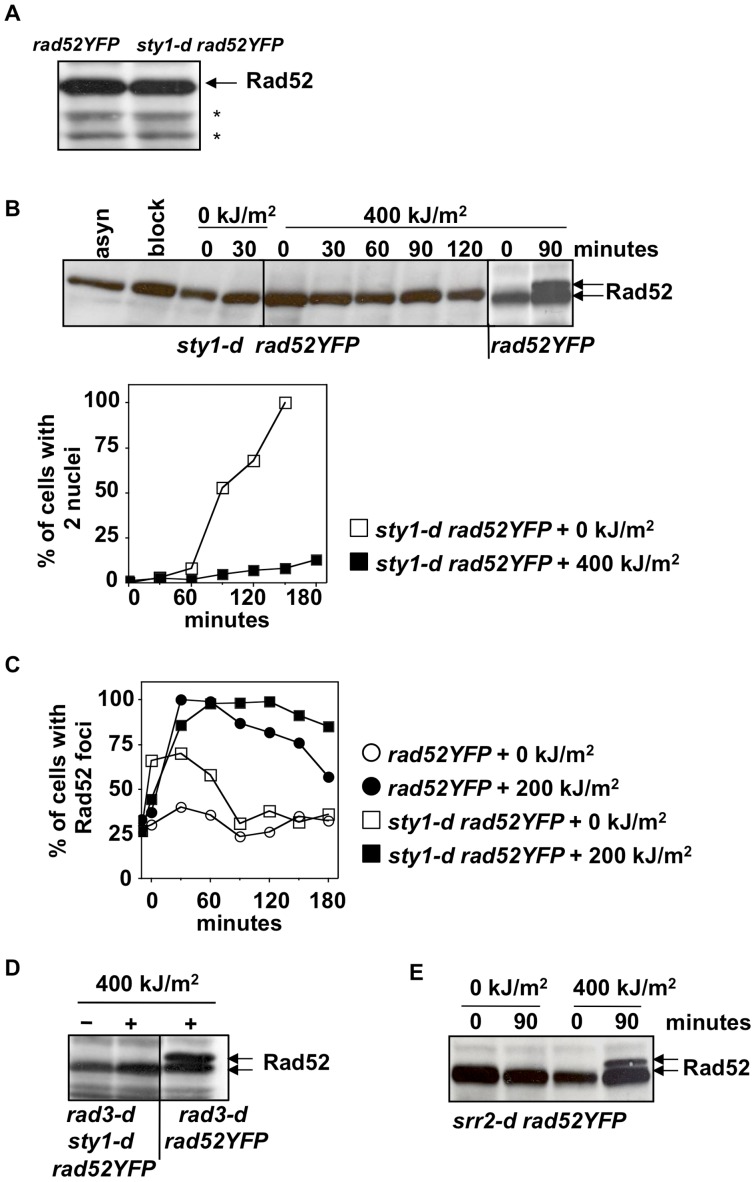
Rad52 phosphorylation upon UVA is Sty1 dependent, but Srr2 independent. (**A**) Rad52 expression detected by anti-GFP antibodies in cells proficient or not for Sty1. Stars indicate aspecific bands serving as loading control. (**B**) Protein extracts at the indicated time points were prepared from cells lacking or not Sty1 and probed with anti-GFP antibodies to detect Rad52 protein (upper panel). Cells were synchronized in early S phase by HU (block) and released into cell cycle after irradiation with either 0 or 400 kJ/m^2^. “asyn” indicates cells in log phase. Delay in cell cycle progression upon UVA was monitored by scoring the percentage of cells with 2 nuclei (lower panel). Time 0 is immediately after irradiation. (**C**) % of cells with Rad52 foci monitored in the indicated strains released from the HU block and exposed or not to UVA radiation. Time 0 is immediately after irradiation. (**D**) Immunoblot with anti-GFP antibodies of protein extracts from asynchronous cells of the indicated strains exposed (+) or not (−) to UVA. (**E**) Immunoblot with anti-GFP antibodies of protein extracts from cells depleted of Srr2 responsive element upon UVA irradiation in early S phase.

Because UVA treated cells experience oxidative stress that elicits activation of the SAPK pathway, we asked if Sty1 kinase would be involved in Rad52 phosphorylation upon UVA. At first, we compare Rad52 expression from exponentially growing cells expressing or not Sty1 (*sty1+ rad52YFP* and *sty1-d rad52YFP* strains) (Figure 4A) and found no differences between the two strains. Then we analyzed Rad52 in *sty1-d rad52YFP* cells exposed to UVA radiation. Rad52 protein was detected prior HU addition (asyn), after 4 hours of HU treatment (block), and every 30 minutes from time 0 till 120 minutes after irradiation. As a control Rad52 protein from *rad52YFP* strain was analyzed at 0 and 90 minutes from release after UVA exposure. As shown in Figure 4B, Rad52 phosphorylation was not detected in UVA-treated cells lacking Sty1, although cell cycle progression was clearly delayed by radiations as judged by the delay in the appearance of cells that have passed mitosis.

Because S phase cells exposed to UVA radiation accumulate Rad52 foci in a dose dependent manner [39] and we showed that Rad52 phosphorylation is Sty1 dependent, we asked if the kinetics of HR foci formation would be affected in *sty1-d* cells. To determine the % of cells having Rad52 foci, *rad52YFP* and *sty1-d rad52YFP* cells were synchronized in early S phase, exposed or not to 200 kJ/m^2^ of UVA and then released into cell cycle. Percentage of cells with Rad52 foci was scored at different time points. Rad52 foci were induced by UVA in both strains, however they persisted longer in *sty1-d rad52YFP* cells if compared to *rad52YFP* strain (Figure 4C). At 180 minutes, about 85% of cells lacking Sty1 had Rad52 foci, in contrast to 55% in control strain (Figure 4C). It is to note that the % of cells with Rad52 foci in *sty1-d* cells released from the HU block without exposure to UVA radiation is greater than in *rad52YFP* cells up to 60 minutes, but then reaches *wt* levels suggesting that the first round of replication after HU is perturbed in cells lacking Sty1.

In addition, as shown in figure 4D, deletion of Sty1 also abolished Rad52 phosphorylation in asynchronous cells exposed to UVA and lacking Rad3 kinase (*rad3-d sty1-d rad52YFP*).

It has been shown in fission yeast that the stress responsive protein Srr2 is phosphorylated in a Sty1 dependent manner upon stress, resulting in nuclear translocation and binding to Rad4 (TopBP1) [51], a scaffold protein that plays a role in both DNA replication and checkpoint response [52]. Thus, we asked if Srr2 might be required for the Sty1 dependent phosphorylation of Rad52 upon UVA. To answer this question we constructed a strain deleted for *srr2* gene and expressing Rad52YFP protein (*srr2-d rad52YFP*). This strain was synchronized in early S phase, irradiated or not with UVA and released into cell cycle. Immediately (0) and at 90 minutes after release, the Rad52 phosphorylation was examined. As shown in Figure 4E, absence of Srr2 protein did not prevent Rad52 phosphorylation.

**Figure 5:**
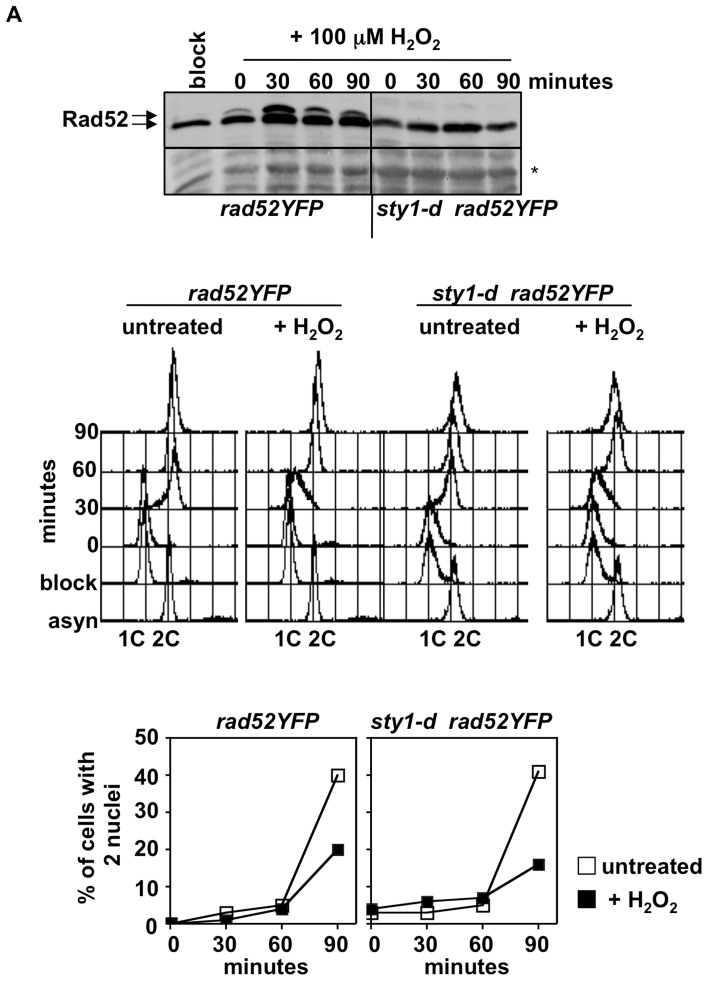
Rad52 phosphorylation upon H_2_O_2_ treatment is Sty1 dependent. **A**) Immunoblot with anti-GFP antibodies of protein extracts from strains *rad52YFP* and *sty1-d rad52YFP* synchronized by HU (block) and released into cell cycle in the presence of 100 mM H_2_O_2_ (upper panel). Delay in cell cycle progression imposed by the treatment was analyzed by FACS analysis (middle panel) and by scoring cells with 2 nuclei (lower panel).

Then we asked if phosphorylation of Rad52 is Sty1 dependent in cells exposed to H_2_O_2_. Treating *sty1-d* cells with 250 mM of oxidant repetitively resulted in almost undetectable Rad52 protein (data not shown), likely due to the Sty1 requirement to support protein synthesis upon exposure to H_2_O_2_ [53]. Thus, we assessed Rad52 phosphorylation upon exposure to lower concentration of oxidant (100 mM) in both Sty1 proficient and deficient cells. 80 and 120 mg of protein extracts were analyzed for *rad52YFP* and *sty1-d rad52YFP* cells, respectively. As shown in Figure 5, Rad52 modification was detected in *rad52YFP* but not in *sty1-d* cells although the treatment with 100 mM H_2_O_2_ delayed cell cycle progression in both strains as judged by FACS analysis and by scoring the percentage of cells that passed mitosis (cells with 2 nuclei).

In conclusion, Rad52 phosphorylation in cells exposed to either UVA or H_2_O_2_ is Sty1 dependent.

### Lack of Sty1 affects spontaneous rate of gene conversion

**Figure 6:**
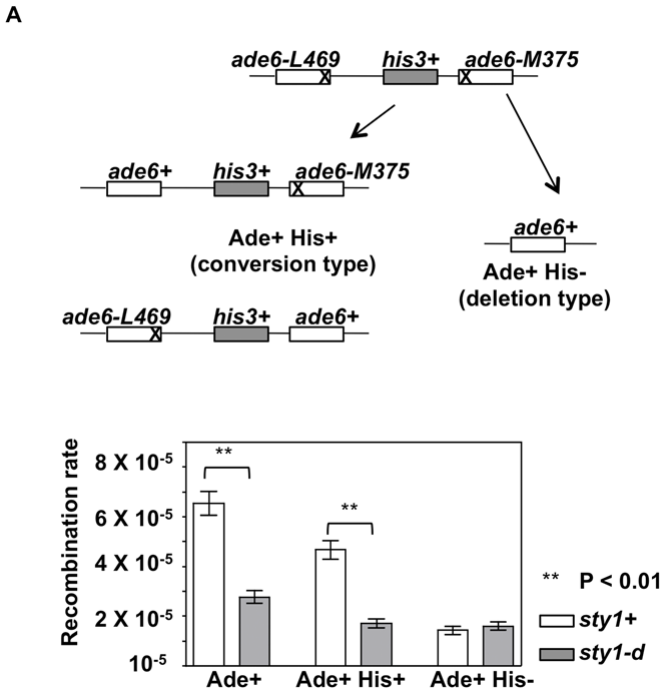
Lack of Sty1 impairs efficient HR. (**A**) Representation of the substrate used to estimate spontaneous recombination rate [54]. Lower panel shows recombination rate of *sty1+* and *sty1-d* cells. Statistically significant differences are indicates by stars.

One possibility explaining the persistence of Rad52 foci in *sty1-d* cells is that HR is less performing in cells lacking the Sty1 kinase. Thus, we assessed the rate of spontaneous HR by fluctuation test in *wt* and *sty1-d* cells using the system described in Osman et al. [54]. This system allows measuring the frequency of recombination between non-tandem hetero-allelic duplications of the *ade6* gene separated by a region of DNA carrying the *his3^+^* gene. With this substrate two classes of Ade+ recombinants can be distinguished: Ade+ His+ resulting from gene conversion and Ade+ His- resulting from deletion of the *his3+* gene located between the two *ade6* alleles (Figure 6A). Because *sty1-d* cells loose viability when reaching saturation in medium containing standard glucose concentrations, HR frequencies were estimated in cultures not yet in stationary phase and used to calculate the rate of spontaneous HR using the MSS-MLE method [43]. As shown in Figure 6B, a significant two fold decrease in the rate of spontaneous HR was unveiled in *sty1-d* cells. While the rate of deletion types (Ade+ His−) was comparable to that of *sty1+* cells, the rate of conversion types (Ade+ His+) in *sty1-d* cells was significantly diminished.

This experiment indicates that Sty1 modulates spontaneous intrachromosomal recombination by promoting one of the mechanisms leading to gene conversion. It also suggests that Sty1 acts on HR also in the absence of exogenous oxidative stress.

### HR at blocked replication forks is diminished in *sty1-d* cells

**Figure 7:**
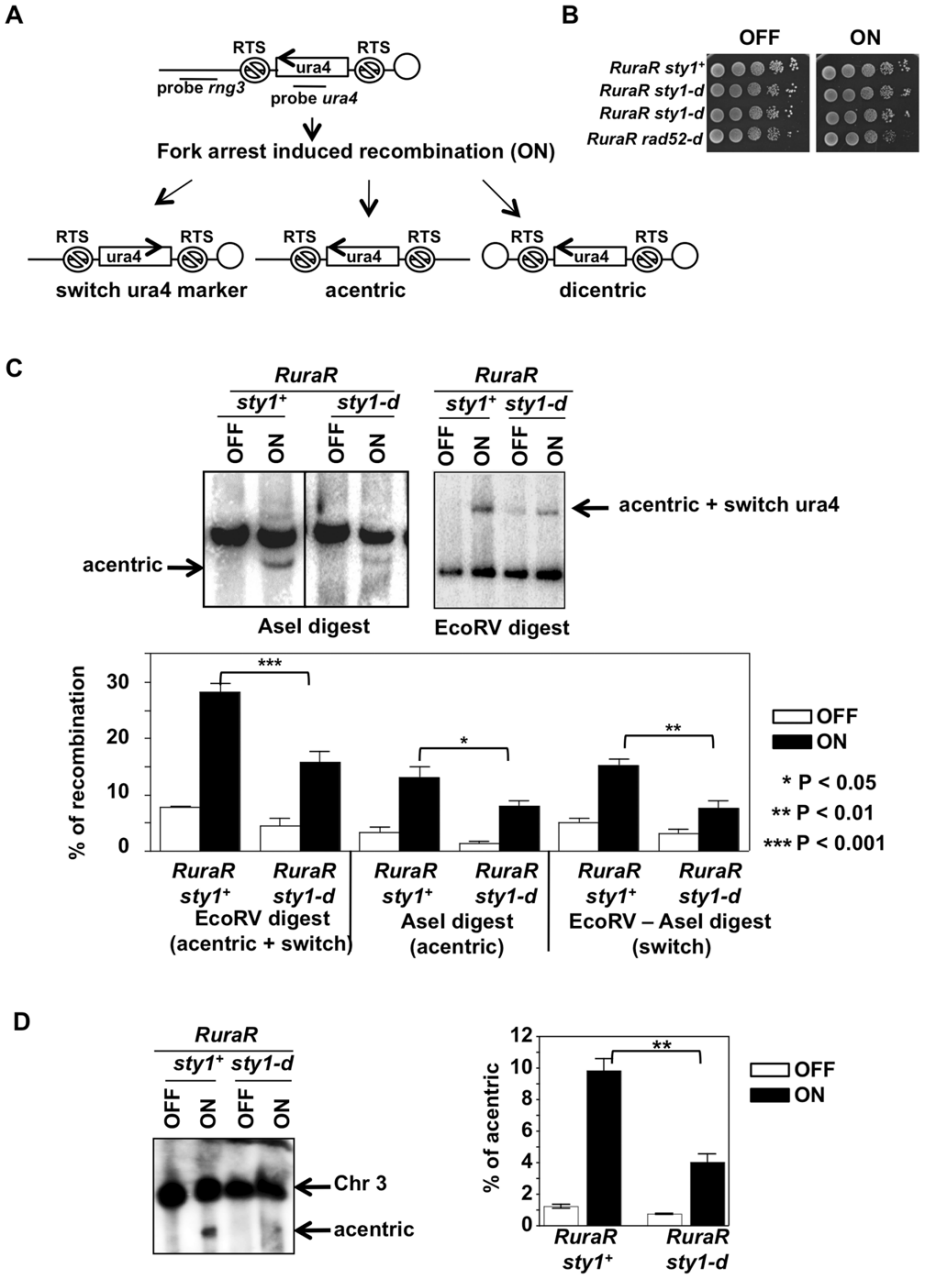
Sty1 is required for efficient HR occurring at blocked replication forks. (**A**) Schema representing the *RuraR* substrate and the possible outcomes after induction of replication block [32]. (**B**) Drop assay of *RuraR sty1+*, *sty1-d* and *rad52- d* cells grown overnight in the absence of thiamine and spotted on medium containing (OFF) or not (ON) thiamine. (**C**) RFLA analysis using *ura4* probe of recombination outcomes in *RuraR sty1+* and *RuraR sty1-d* cells under conditions of induction (ON) or not (OFF) of replication fork arrest. The % of recombination was estimated from three independent experiments. Stars indicate statistically significant differences. (**D**) Analysis by PFGE of acentric chromosome 3 formation upon induction of replication fork arrest (ON) using *rng3* probe and quantification from two independent experiments. Stars indicate statistically significant differences.

The results obtained using UVA and H_2_O_2_ suggest that the Sty1 pathway impinges on HR at least in cells undergoing S phase. We took advantage of the *RuraR* inducible system (Figure 7A) described in Lambert et al. [32] to assess recombination induced by replication fork arrest. This system allows replication fork to be blocked at the RTS1 fork barrier near the *ura4+* gene (*RuraR* substrate) on chromosome 3. Induction of replication fork arrest is obtained by controlled expression of Rtf1 protein that binds RTS1 sequence creating a barrier to fork progression: expression is shut down in medium containing thiamine (OFF) and is induced in medium depleted of thiamine (ON) leading to fork arrest. Restart of arrested replication forks requires HR and, occasionally, fork arrest induced recombination will result in genome rearrangements (Figure 7A) [32]. A strain deleted for *sty1* and containing the *RuraR* substrate and the inducible *rtf1+* gene was constructed (*RuraR sty1-d*). Growth rate of this strain was comparable to the one of *RuraR sty1+* (not shown). Survival of *RuraR sty1-d* strain was compared to the *RuraR sty1+* and to *RuraR rad52-d* by growing cells over night in the absence of thiamine (ON) and spotting serial dilutions on plate containing (OFF) or not (ON) thiamine. The *RuraR sty1-d* strain behaved as *RuraR sty1+* (Figure 7B).

Restriction fragment length analysis (RFLA) showed that rearrangements were induced by replication forks arrest (ON) in both *sty1+* and *sty1-d* strains. However, *RuraR sty1-d* cells had decreased levels of *ura4+* marker switch and of acentric chromosome formation if compared to strain of reference. Quantification from three independent experiments indicated a significant two fold decrease in % of HR occurring at blocked replication forks in *RuraR sty1-d* cells (Figure 7C). The reduced formation of acentric chromosome in *RuraR sty1-d* cells was confirmed by PFGE where chromosome 3 containing the substrate was identified by hybridizing with *rng3* probe (Figure 7D).

Thus, in line with the results of the experiment measuring spontaneous recombination rates, HR occurring at blocked replication forks is less efficient in cells with disabled SAPK pathway suggesting that it impinges on HR even in the absence of external oxidative stress. Both the lower spontaneous HR rate and the lower percentage of HR at blocked replication forks are consistent with the suggestion that HR is less performing in *sty1-d* cells exposed to UVA, where Rad52 foci persist longer.

### Rad52 is constitutively phosphorylated in a Sty1 dependent manner in cells lacking Rhp51

**Figure 8:**
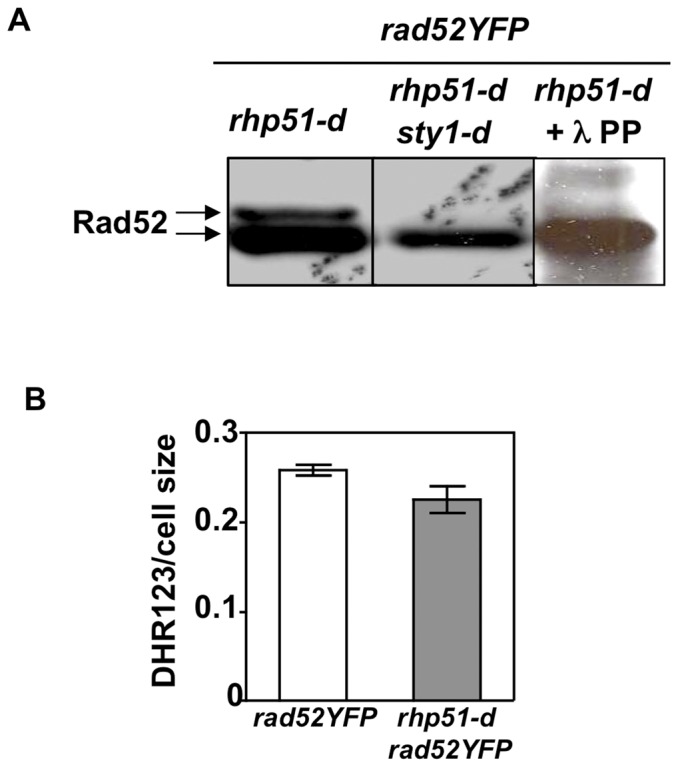
Sty1 dependent Rad52 phosphorylation is constitutively detected in cells lacking Rhp51. (**A**) Immunoblot with anti-GFP antibodies of protein extracts from strains *rhp51-d rad52YFP* (left lane) and *rhp51-d sty1-d rad52YFP* (middle lane). The change in mobility shift of Rad52 protein in *rhp51-d rad52YFP* cells is abolished by treatment with l PP (right lane). (**B**) Measurement of intracellular ROS levels using the fluorescent probe DHR1,2,3 in *rad52YFP* and *rhp51-d rad52YFP* cells. Bar represents the SEM of two independent experiments.

The results presented above indicate that Sty1 modulates HR even in the absence of exogenous oxidative stress and that Rad52 phosphorylation might be implicated in recombination processes. However, Rad52 phosphorylation was not observed in cells not exposed to oxidative stress. As we suggest in the discussion, a Sty1 dependent phosphorylation of Rad52 might be difficult to detect in untreated cells because of low basal levels of HR. Thus, we looked at Rad52 phosphorylation in cells lacking the Rhp51 recombinase (*rhp51-d rad52YFP*) where early steps of HR are impaired [35]. We found that a slower migrating band of Rad52, which can be reversed by lPP treatment, was detected in cells lacking Rhp51 in the absence of any external insult (Figure 8A, first and third lanes), indicating that Rad52 is constitutively phosphorylated when early step of HR is prevented. More importantly, deletion of *sty1* gene abolished Rad52 phosphorylation in *rhp51-d* cells (Figure 8A, second lane). In addition, the intracellular concentration of ROS in *rhp51-d rad52YFP* cells, measured using the redox sensitive fluorescent probe DHR123, was similar to the control strain (Figure 8B), indicating that intrinsic oxidative stress is unlikely what is triggering the Sty1 dependent Rad52 phosphorylation in *rhp51-d* cells.

Thus, these results support the notion that Sty1 pathway impinges on HR through phosphorylation of Rad52 even in the absence of treatments inducing oxidative stress.

## Discussion

Here we present evidence that the SAPK pathway phosphorylates Rad52, a protein central to HR repair, when cells are subject to oxidative stress either by exposure to UVA or to H_2_O_2_. We also show that in the absence of exogenous treatment the Sty1 kinase promote spontaneous mitotic gene conversion and recombination induced by replication fork arrest. Importantly, we show that cells lacking the Rhp51 recombinase display constitutive Rad52 phosphorylation that is also dependent on Sty1 kinase.

A role for fission yeast Sty1 in HR has been shown for meiotic hotspot recombination where Sty1 regulates in a phosphorylation-independent manner the positioning of transcription factor Atf1 at the M26 hotspot. The Sty1 role in meiotic hot spot recombination together with the observation that it is dispensable for basal meiotic recombination, suggest that Sty1 does not regulate general key elements of recombination during meiosis [55]. Rather, chromatin remodeling in meiosis triggered by Sty1 activation in response to nitrogen starvation allows large protein complexes, such as the HR machinery, to be recruited at the hotspot recombination sites [56].

It has also been proposed that all three major MAPK pathways affect mitotic HR in human malignant glioma, but the molecular basis of the MAPK impact on HR remain unknown, and it was concluded, based on the use of SAPKs inhibitors, that ERK and JNK positively regulate HR while p38 has an opposite impact on it [57].

In the first part of our work we studied Rad52 modification in replicating cells exposed to different exogenous treatments. Sty1 dependent Rad52 phosphorylation was observed upon UVA and H_2_O_2_ treatments in replicating cells. Both agents induce a delay in DNA replication progression, but this is not likely the reason leading to Rad52 phosphorylation, since we show that UVC radiation, which also delays replication, did not induce Rad52 phosphorylation. In addition, induction of replication stress in S phase by CPT that leads to requirement for HR also did not result in Rad52 phosphorylation, suggesting that collapse of replication forks because of replisome loss is not a requirement for Rad52 modification. Rather, Rad52 phosphorylation correlated with ROS production since concomitant treatment of cells with oxidant and antioxidant reduced the replication delay and Rad52 phosphorylation. However, Rad52 phosphorylation is not required for replication delay upon oxidative stress, since cells lacking Sty1, and thus Rad52 phosphorylation, still delay replication (this work and [39]).

These observations suggest that the Sty1 dependent phosphorylation of Rad52 is induced by oxidative stress rather than by DNA lesions. Though, we didn't observed Rad52 phosphorylation after UVC although it has been shown that UVC radiation induces the Sty1 oxidative stress response explaining the hypersensitivity to UVC of *sty1-d* cells [58]. This discrepancy might outline the greater requirement for HR repair after UVA and H_2_O_2_ than after UVC radiation or might reflect different responses to different levels of oxidative stress.

According to our results, the response to oxidative stress is required to promote HR since Rad52 foci persist longer in cells lacking Sty1 where Rad52 is not phosphorylated. We do not know if Rad52 is a direct substrate of Sty1 and we cannot exclude that another kinase acting downstream of Sty1 phosphorylates Rad52 in the nucleus. Although we didn't succeed in identifying by 2-dimensional electrophoresis followed by mass spectrometry the sites of phosphorylation because the Rad52YFP protein failed to separate by isoelectric focusing under different experimental conditions, we ruled out the contribution of kinases Chk1, Cdc2, Rad3 and Tel1 in Rad52 phosphorylation.

We have shown that the rate of spontaneous HR is reduced in *sty1-d* cells that are not exposed to exogenous oxidative stress, and that loss of recombinants is at charge of gene conversion types. Differently from meiotic recombination, mitotic HR is rarely associated with crossovers and different published data suggest that SDSA (Synthesis-Dependent Strand Annealing) is the major non-crossovers mechanism in DSBs repair during mitosis. In SDSA as well as in DSBR (DSB Repair) model, after resection at DSB, a 3′ single strand DNA tail is generated that will be coated by Rad51 through a process requiring Rad52. This permits strand invasion and D loop formation allowing DNA synthesis using the homologous unbroken DNA sequence as a template. In SDSA, differently from DSBR, the D loop is displaced and newly synthesized DNA pairs to the other end of DSB. If the homologous sequences are heteroallelic, then the paired DNA will contain a mismatch. Alternatively, a mismatch between the invading strand and the unbroken molecule is formed during D loop extension. Mismatches are detected by the mismatch repair system (MMR) that will either reject the invading strand or correct the mismatch possibly leading to gene conversion.

Similarly, the recombination dependent genome rearrangements upon replication fork arrest in the *RuraR* system result from a mechanism of template exchange where the newly synthesized strand at blocked replication fork will engage in formation of D loop by pairing on the homologous noncontiguous sequence [44]. Since we observed in cells experiencing exogenous oxidative stress a correlation between absence of Sty1 dependent Rad52 phosphorylation and persistence of Rad52 foci, it might be that lower levels of rearrangements at blocked replication forks as well as reduced spontaneous gene conversion in *sty1-d* cells result from incapacity to form a stable D loop. According to this hypothesis, a Sty1 dependent phosphorylation of Rad52 should occur also in cells not exposed to oxidants, however it might be difficult to detect it because of low basal levels of HR. Consistently, we didn't observed Rad52 phosphorylation using the *RuraR* system where one replication fork per cell is blocked (not shown). Alternatively, Rad52 phosphorylation by Sty1 occurs only upon Sty1 activation by oxidative stress while basal levels of Sty1 affect spontaneous HR and HR at blocked replication forks without Rad52 modification. However, we have shown that blocking D loop formation by inactivation of Rhp51 recombinase results in Sty1 dependent Rad52 phosphorylation. Because *rhp51-d* cells don't seem to have an altered redox state as judged by measurement of intracellular ROS, it is unlikely that Rad52 phosphorylation in this genetic background results from activation of Sty1 by oxidative stress. Nevertheless, our results point to a possible role of phosphorylated Rad52 in early step of HR. The question rises about the signal bringing cytosolic Sty1 to promote HR in the nucleus of cells lacking Rhp51 or bearing either the intrachromosomal recombination substrate or the *RuraR* system.

In conclusion, we bring evidence that SAPK pathway influences HR, a DNA repair pathway with implications in both protecting and promoting genome rearrangements that can drive oncogenesis.
